# Understanding Physical Activity in Patients With Metastatic Breast Cancer: An Analysis Rooted in the Theory of Planned Behaviour

**DOI:** 10.1002/pon.70457

**Published:** 2026-04-16

**Authors:** Esther C. E. de Jongh, Johanna Depenbusch, Anne May, Anouk Hiensch, Yvonne Wengström, Jon Belloso, Milena Lachowicz, Lonneke van de Poll‐Franse, Karen Steindorf, Martijn M. Stuiver

**Affiliations:** ^1^ Department of Psychosocial Research and Epidemiology Netherlands Cancer Institute Amsterdam the Netherlands; ^2^ Division of Physical Activity Cancer Prevention and Survivorship German Cancer Research Center (DKFZ) and National Center for Tumor Diseases (NCT) Heidelberg Germany; ^3^ Julius Center for Health Sciences and Primary Care University Medical Center Utrecht Utrecht University Utrecht the Netherlands; ^4^ Division of Nursing Department of Neurobiology Care Sciences, and Society Karolinska Institute, and Karolinska Comprehensive Cancer Center Karolinska University Hospital Stockholm Sweden; ^5^ Gipuzkoa Cancer Unit OSID‐Onkologikoa Biogipuzkoa Osakidetza San Sebastián Spain; ^6^ Department of Oncology and Radiotherapy Medical University of Gdańsk Gdańsk Poland; ^7^ Tilburg School of Social and Behavioral Sciences Tilburg University Tilburg the Netherlands; ^8^ Epidemiology and Data Science Amsterdam UMC location University of Amsterdam Amsterdam the Netherlands

**Keywords:** behavioural pathways, cultural differences, exercise barriers, exercise intentions, health behaviour, metastatic breast cancer, physical activity, theory of planned behaviour

## Abstract

**Objectives:**

This study aims to improve understanding physical activity behaviour in patients with metastatic breast cancer, based broadly on Ajzen's Theory of Planned Behaviour (TPB). We assessed the role of different pathways in the TPB and of cancer specific barriers to find points of leverage for improving exercise support and behaviour in this vulnerable population. In addition, we explored international differences in the model's pathways.

**Methods:**

We conducted an international survey (*n* = 420) amongst patients with metastatic breast cancer, including items assessing attitude, injunctive subjective norm, intention, perceived behavioural control (PBC), and self‐reported physical activity. Additionally, we examined the relative importance of health‐related barriers on motivational pathways.

**Results:**

Model fit indices indicated a reasonable fit for the TPB (cfi = 0.98, rmsea = 0.08) with small (standardized absolute coefficients 0.13–0.30) but statistically significant associations in the expected directions according to the theoretical model, except for subjective norm (*β* = 0.05). The interaction of PBC with intention was statistically significant, but adding this term diminished overall model fit (cfi = 0.85, rmsea = 0.13). The presence of health‐related barriers impacted behaviour (*β* = −0.19) whilst acting as a mediator for PBC on intention. International differences in the strength of model pathways suggest cultural variation.

**Conclusions:**

Findings support the applicability of the TPB for understanding and supporting physical activity behaviour in patients with MBC. Targeting perceived behavioural control may be especially effective in overcoming health‐related barriers in this population. Additionally, improving affective and instrumental attitude towards physical activity could enhance intervention outcomes.

## 1 Background

Metastasized breast cancer (MBC) is increasingly managed as a chronic condition, as the median survival rate of this patient population is counted in years rather than months, unlike other metastatic cancers [1]. Accordingly, its treatment focuses on supporting patients' ability to live with the disease whilst maintaining quality of life, rather than viewing outcomes as strictly curative or palliative [2, 3]. In parallel, patients with MBC are expected to implement self‐management of their symptoms and wellbeing into their lives [4, 5, 6]. Opportunities to engage in behaviour that contributes to positive health outcomes may also offer a sense of agency and control in a context where certainty is not guaranteed [3, 7, 8, 9].

Although evidence has primarily focussed on patients treated with curative intent, emerging research demonstrates that the benefits of exercise also apply to patients with metastatic disease [10, 11, 12, 13, 14, 15].

Despite its benefits, physical activity (PA) levels often decline during treatment due to common side effects (such as fatigue or pain) or logistical barriers (such as the interference of medical procedures or lack of access to appropriate facilities) [16, 17, 18, 19, 20, 21]. Fatigue, weakness, and lack of access to (specialized) exercise facilities are amongst the most frequently reported barriers to consistent physical activity in patients with MBC, and the international PERSPECTIVE study (primary study described in the Methods section) revealed this group is in need of support [22, 23, 24].

The Theory of Planned Behaviour (TPB) describes the underlying pathways for engaging in an intended behaviour and has been effectively used as a framework to understand and support health behaviour in different populations [25, 26, 27, 28, 29]. The explanatory power of the TPB has been demonstrated in several cancer populations, where it has successfully contributed to the development of interventions promoting PA in the past. [10, 30]^,^ The TPB has been studied in the context of chronic illness, yet it remains necessary to determine its utility for PA behaviour in patients with MBC specifically, as the relative importance of each path in the TPB shifts depending on the population to which it is being applied (a summary of the current state of the TPB is provided in Supplementary File 1) [26, 30, 31, 32, 33, 34, 35]. As patients with MBC face distinct challenges and experience a shift in their perspective, these pathways may be affected differently in this population [25, 36, 37].

The data from the PERSPECTIVE study offers opportunities to clarify how the TPB might help understand PA behaviour of patients with MBC, as the survey contains items rooted in this model. Understanding the strength of the TPB pathways in this population could provide clinicians with actionable insights to improve their quality of life. Therefore, the **first objective** of this study is to assess whether the TPB can be used in the MBC population, and if so, which variables have the strongest association. In doing so, this study will yield insights on which paths are fruitful in the promotion of PA in women with MBC. The **second objective** is to report on the relative importance of (external) barriers on intention and behaviour. These barriers—often related to disease‐ and treatment‐specific health issues—may be amenable to intervention, thereby informing the implementation of exercise interventions for this population in the future [38]. Finally, the concepts measured in the TPB also relate to how individuals think about themselves, their capabilities to perform tasks, and how they handle their illness [39]. Cultural psychologists agree on the fundament that the self, and its formulation of thought and behaviour in daily practise, is inherently shaped by culture [40, 41, 42]. Through interaction with social others and prevalent belief systems, culture guides individuals in the values and tendencies they develop, on both subconscious and conscious levels. As a result, self‐regulation practices are likely to—at least in part—reflect values and attitudes specific to a culture [43, 44, 45]. Studies comparing self‐management strategies between advanced cancer from different cultural backgrounds are limited, but notable differences have been identified [46, 47, 48, 49]. Similarly, it is plausible that measured concepts in the TPB, and their subsequent effects on intention and PA behaviour, might vary between the five patient nationalities in this study. Taking advantage of our international sample, the **third objective** of this study is to explore how cultural differences might affect the strength of different pathways in the TPB in the five European subsamples.

## 2 Methods

The data used for this study were collected through the PERSPECTIVE survey study as part of the EU‐funded PREFERABLE project (grant agreement No. 825677). The methods for that study have been published elsewhere [22]. In short, the survey was distributed to patients with MBC in 5 European countries: the Netherlands, Poland, Sweden, Germany and Spain, via direct recruitment in the hospitals of the PREFERABLE consortium and adjacent hospitals. A small proportion of respondents (6%) were recruited via open online recruitment strategies [22]. Besides the survey's primary aim of collecting views on barriers and facilitators of exercise, the survey included questions on cognitive behavioural variables based on the Theory of Planned Behaviour [25]. The questions were first developed in English and then translated to each of the respective languages using a rigorous forward‐backward translation procedure [50]. Official translations were used for validated questionnaires.

### 2.1 Ethical Approval

Participating study centres obtained ethical approval from their respective Ethics Committee and informed consent was obtained from all patients.

### 2.2 In‐ and Exclusion Criteria

Data from 420 respondents to the survey was available as the source data for the current objectives. The inclusion criteria of the PERSPECTIVE study comprised: a diagnosis with histologically confirmed MBC, aged ≥ 18 years, an Eastern Cooperative Oncology Group (ECOG) performance status score of ≤ 2 (indicating that respondents ranged from being fully active to ambulatory and capable of self‐care but unable to work), a sufficient command of any of the languages in which the questionnaire was available. Exclusion criteria included a life expectancy of < 6 months, patients who were not able to perform basic activities of daily living, or had cognitive problems that precluded the completion of a questionnaire [22].

### 2.3 Included Variables

For the different TPB constructs, we used either previously applied and validated scales, or developed items in accordance with the recommendations from previous studies: for patients' overall attitude, patients' exercise competence, possible barriers and facilitators, and patients' expectations (Supporting Information S2: Appendix A) [51, 52, 53, 54, 55].

*Attitude* towards PA behaviour was measured with 6 items, of which 3 items encompass affective attitude, describing PA as enjoyable, fun, and easy. The other 3 items encompass instrumental attitude, through describing PA as useful, beneficial, and sensible. These items were measured using a 7‐point Likert scale (−3 to 3). The mean scores of affective attitude and instrumental attitude were calculated for the analysis.

*Subjective norm* was measured to reflect the propensity to conform to health care professionals' advice on PA, thusly measuring injunctive subjective norm. The item consisted of a single statement (i.e., ‘I would exercise if my doctors and other health care professionals encourage me to do so’) measured using a 7‐point Likert scale.

*Perceived behavioural control* (PBC) was operationalized through the Exercise Self‐Efficacy Scale, which is grounded in Bandura's Self‐Efficacy theory and commonly used to represent PBC. The scale includes 9 items that present hypothetical situations of low mood, stress, time constraints, lack of social support, low outcome expectations, physical complaints (stiffness or soreness), poor weather and low enjoyment [56, 57]. Respondents were asked how confident they were that they could exercise in these 9 respective circumstances, using a 5‐point Likert scale ranging from ‘not at all confident’ to ‘extremely confident.’ The mean score was used in the analysis.

*Intention* was expressed as a single item: *Did you intend to start or continue exercising in the month prior to the coronavirus outbreak?*, with a 7‐point Likert scale ranging from ‘definitely yes’ to ‘definitely no’.

*Behaviour* was derived from 4 items based on the Godin Leisure Time Questionnaire, which queried the number of average weekdays on which respondents engaged in light, moderate and strenuous PA, respectively, as well as the number of minutes per day for each PA category [58]. From these questions, we calculated the total minutes of at least moderate intensity PA during a week.

*Barriers* included health‐related barriers that affected individuals' perception of their ability to perform the intended PA behaviour [53, 55]. This included feeling too weak, comorbidities, pain, shortness of breath, tiredness, fear of falling, and being unsure of how to begin or how much exercises should be done. Each barrier was scored on a 5‐point Likert scale ranging from ‘not at all’ to ‘very much’, and the total score was used in the analysis (possible range 0–32).

### 2.4 Statistical Methods

Descriptive statistics were calculated to summarize respondent characteristics. Variables measured on an interval scale were presented as mean and standard deviation, or median and interquartile range in case of a skewed distribution. Categorical variables were summarized as absolute and relative frequencies.

For the first objective of the study, we fitted a path model representing the TPB using the Lavaan package in R (version 4.3.3) [59]. Considering the variety of barriers queried in the survey, we first conducted a principal component analysis to organize them into relevant subgroups. Cronbach's alpha was calculated to determine the internal consistency reliability for barriers. Scale coverage was calculated for each construct as the percentage of the possible score range relative to the observed score range. We applied a square root transformation for PA behaviour. All variables were standardized prior to the analyses, to account for different score ranges and variances, and allow direct comparisons. Following Cohen's guidelines, we interpreted coefficients of 0.2 as small, 0.5 as moderate, and 0.8 as strong [60]. Paths were modelled as regressions, whilst accounting for the covariance of the explanatory variables. The root mean squared error of approximation (rmsea) and comparative fit index (cfi) were used to evaluate overall model fit. Values for rmsea < 0.05 indicate good fit and values ≤ 0.08 < 0.10 acceptable fit, as popularized by Browne & Cudeck [61]. For cfi, values closer to 1 indicate a better fit, with ≥ 0.95 indicating a good fit.83 We adopted a conservative approach for adding or removing paths, by only considering paths that made theoretical sense, had modification indices (MI) > 10, and improved the model fit (according to cfi and rmsea). In the models exploring how barriers fit into the model, we only considered MIs that related to the barrier paths. Models with added paths were compared using the Bayesian information criterion (bic, lower value indicating better fit) and cfi, to choose the final model best representing the data. For the final model, we confirmed model assumptions (i.e., normal distribution of the residuals).

Inter‐country differences were explored by fitting the models to subgroups representing the respective countries (Figure 2). Estimates of this analysis and their 95% confidence intervals were compared based on a forest plot. We did not include our Swedish sample when speculating about inter‐country differences, due to its wide CI's, likely a result of the small subsample size (*n* = 48).

### 2.5 Handling of Missing Data

Missing data on minutes per day of PA in any category were directly imputed as 0 if the days/week variable for that PA category was 0, and vice versa. To deal with remaining missing data, we used full information maximum likelihood estimation (FIML) when fitting the models.

### 2.6 Sensitivity Analyses

We performed three sensitivity analyses: one using only time spent at PA of moderate or higher intensity, a second excluding Swedish participants because of a slightly different recruitment strategy used (i.e., patients were more often recruited from the source population for the PERSPECTIVE‐EFFECT RCT) [10], and a third with imputed data for all remaining missing variables (i.e., after direct imputation of the physical activity data as described above), using k‐nearest neighbours imputation.

## 3 Results

Data from 420 respondents were available for this study. Overall, 416 respondents were female and the mean age was 56.5 (SD = 9.2). Full characteristics are presented in Table 1.

**TABLE 1:** Descriptives of survey participants and scale coverage.

	N or M		% or SD	
Age^a^	56.5		9.2	
Female^b^	416		99.0	
BMI^b^				
Underweight	19		4.6	
Normal weight	193		46.3	
Overweight	132		31.7	
Obese	73		17.5	
Country of residence^b^				
Sweden	48		11.4	
Spain	99		23.6	
Poland	64		15.2	
The Netherlands	111		26.4	
Germany	98		23.3	
Marital status^b^				
Married/living with a partner	299		71.2	
Divorced/Widowed/Single	121		28.8	
Highest educational level^b^ ^,^ ^c^				
Academic education	177		42.2	
Higher education	115		27.4	
Middle education	103		24.6	
No or basic education	24		5.7	
Current employment status (paid work)^b^				
Employed	186		44.3	
Not employed	234		55.7	
Physical activity behaviour in minutes				
Total time spent in any PA behaviour	120		0–300^d^	
Total time spent in moderate and/or vigorous PA behaviour	0		0–120^d^	

**Scale**	**Median**	**Range**	**Scale coverage**	**Reliability** (α)
Instrumental attitude	3	−3–3	83%	0.83
Affective attitude	2	−3–3	100%	0.79
PBC/SE	2	0–4	80%	0.89
Subjective norm	3	0–4	80%	N/A
Intention	6	0–6	100%	N/A
Barriers	8	0–32	97%	0.83

^a^ Displayed as mean (M) and standard deviation (SD).

^b^ Displayed as counts (N) and percentage (%).

^c^ Academic education: bachelor degree or higher (according to Europe‐wide Bologna process); higher education: degree qualifying for university; middle education: degree qualifying for further vocational training.

^d^ Interquartile range.

### 3.1 Comparing TPB Model Fits

**FIGURE 1:**
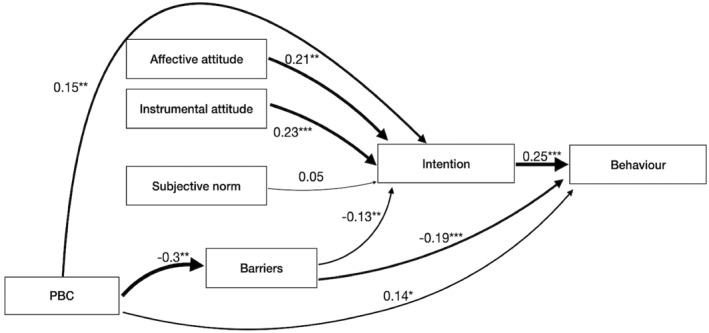
Strengths of associations within the Theory of Planned Behaviour (TPB) model. Arrow thickness represents the magnitude of relationships between constructs, with thicker arrows indindicating stronger associations.

We fitted both path models, representing the TPB and the TPB with intention‐PBC interaction, respectively, and compared the fit indices between the two. Model fit indices indicated a reasonable fit of the TPB to the data (cfi 0.98, rmsea 0.077), with all associations in the expected directions. Most associations were small, however, and the influence of subjective norm on intention was negligible and not statistically significant (Figure 1). When applying the TPB with intention‐PBC interaction, the moderator coefficient was indeed statistically significant, yet model fit indices indicated a weaker fit to the data compared to the TPB: cfi 0.914, rmsea 0.103. Therefore, we continued with the TPB.

Based on the principal component analysis, barrier items were grouped into three subscales, representing practical barriers (alpha = 0.74), competing demands (alpha = 0.68), and health‐related barriers (alpha = 0.79), respectively. Adding all barrier subscales as a predictor of PA behaviour strongly reduced model fit (cfi 0.595, rmsea 0.17). Practical barriers and competing demands appeared unrelated, whereas health‐related barriers demonstrated a significant but weak association with PA behaviour. We therefore only used the health‐related barriers subscale in subsequent models.

The final model largely confirmed the paths hypothesized by the TPB: Both affective and instrumental attitudes were independently, positively and significantly associated with intention. Both higher intention and PBC are linked to increased PA behaviour, in line with earlier studies in cancer survivors [26, 62]. The model further indicates that higher PBC is associated with fewer perceived health‐related barriers, and that there is a significant inverse association between health‐related barriers and intention as well as behaviour (Figure 1). Subjective norm has a small and non‐significant relationship with intention. The rmsea of this model was 0.129 and the cfi 0.91. Sensitivity analysis yielded similar conclusions (data not shown).

### 3.2 Differences per Country

**FIGURE 2:**
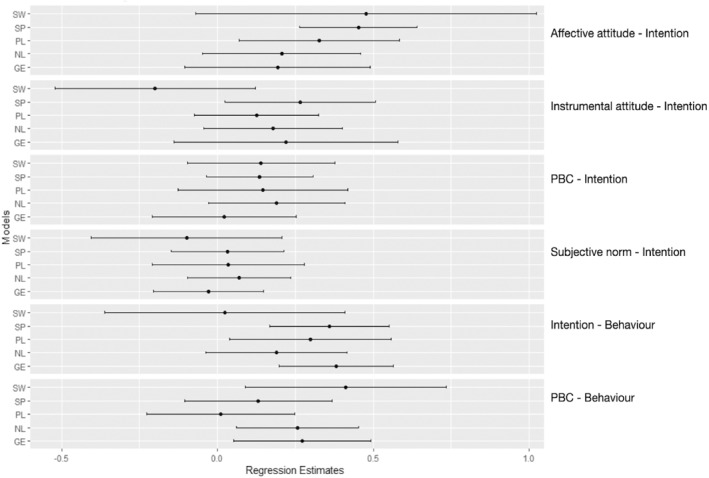
Forest plot of regression estimates per country. GE, Germany; NL, The Netherlands; PBC, Perceived behavioural control; PL, Poland; SP, Spain; SW, Sweden.

The differences in regression estimates between populations can be observed in Figure 2, potentially reflecting methodological differences or cultural variations. Affective attitude has a stronger effect on intention in our Spanish sample, compared to our samples in the Netherlands and Germany. In addition, PBC does not seem to influence behaviour in our Polish sample.

## 4 Discussion

Overall, this study demonstrates applicability of the TPB to understand PA behaviour amongst patients with MBC. Both affective and instrumental attitudes were positively associated with intention, in agreement with previous studies demonstrating that attitudes are important in predicting physical activity intentions [63, 64, 65, 66]. Instrumental attitudes are likely influenced by outcome expectations, whilst patients with MBC are not always aware of how exercise might benefit their symptom control [22, 67].

We did not find a significant association between (injunctive) subjective norm and intention. In part, this could be due to the limited scope of this measure in our study, measuring normative beliefs only through advice of health care professionals as an important other. Although Rodrigues et al. examined subjective norm in relation to behaviour rather than intention, our findings similarly suggest that subjective norm may play a limited role, contributing to the broader uncertainty surrounding its influence [66]. However, our finding is still at odds with the study by Courneya & Friedenreich, which shows physician approval does predict intention in patients undergoing breast cancer treatment [26]. The weak influence of subjective norm in our sample of MBC patients could also reflect a mentality shift in taking responsibility of one's health due to their disease stage. Future studies could further explore this possibility. Nevertheless, previous studies do show larger effects of attitude on intention compared to subjective norms on intention (irrespective of the type of behaviour being assessed) [64, 68, 69]. In addition, a study by Conner et al. demonstrates that injunctive norms are only significant predictors of intention in health protection behaviours [70], such as getting vaccinated or wearing seatbelts and helmets, whereas physical activity for patients with MBC might be considered a health *promoting* behaviour.

Our data highlight a strong inverse association between PBC and health‐related barriers, as well as a relative inverse impact of health‐related barriers on both intention and behaviour. This aligns with the underlying assumptions in the TPB: Individuals with higher beliefs in their control over performing the intended behaviour will cite fewer barriers [25, 27, 71].

Lastly, the TPB with intention‐PBC interaction proposed a moderating effect of PBC on the intention‐behaviour relationship. Although we did find a statistically significant moderating effect, which is in line with the findings of a recent meta‐analysis [64], the TPB excluding this interaction showed a better fit to the data. As we valued sparsity over including all statistically significant variables for this study, we did not retain the interaction variable.

### 4.1 International Differences

As part of our third objective, we leverage our international sample and assume cultural differences between countries, treating nationality as a proxy for cultural identification [42]. This allows us to explore cultural differences in model pathways that might play a role in PA behaviour.

We observe a stronger effect of affective attitude on intention in our Spanish sample compared to the Dutch and German samples (Figure 2). One prominent cultural difference between Spain and the latter two countries can be understood through Hofstede's theorization on individualism and Markus & Kitayama's work on self‐construal [42, 72]. With a lower individualism score, Spain is considered a more interdependent society compared to the more independently construed German and Dutch societies, which may lead to different approaches to decision‐making than the more rational styles typically observed in independent cultures [42, 72, 73, 74]. In interdependent contexts, emotional, relational, and situational considerations may weigh more heavily in decision making than purely rational evaluations, which could explain the relative strength of affective attitude in our Spanish population [75]. However, further research is needed to study this relationship in context of health‐promoting behaviours and explore how different modes of self‐construal may influence pathways in the TPB.

PBC does not have an effect on behaviour in our Polish sample, suggesting that cultural differences may play a role in PBC between populations [76]. A study by Kosińska & Nowak found that self‐efficacy (used to measure PBC) had a negative effect on positive health behaviours in Polish students, as opposed to the positive effect of self‐efficacy on health behaviours in their Spanish peers [77]. Drawing on Markus and Kitayama's framework of independent versus interdependent self‐construal, the Polish sociocultural context may promote a more interdependent orientation, where the experience of agency is affected by social expectations or the need to conform [42, 44, 78]. In addition, the historical presence of external influences in Polish political and cultural narratives might negatively affect the internalization of control and induce cautious decision‐making [78, 79]. This could explain how Polish samples are less likely to internalize a sense of control or trust in their ability to influence outcomes, weakening the connexion between self‐efficacy (PBC) and PA behaviour.

### 4.2 Study Limitations

Several limitations to this study should be acknowledged. The items used for this study were part of a of a larger survey, which implies a trade‐off between completeness and respondent burden. In particular, subjective norm was only assessed through injunctive normative beliefs, using a unidimensional scale measuring the beliefs of health care professionals. However, Fishbein and Ajzen's most recent construct includes two subdomains: injunctive normative beliefs and descriptive normative beliefs [80]. As no significant associations were found, we do not think the limitation of this variable impacted the interpretation of our findings. Intention was also measured using a single item, limiting the reliability of this construct. Moreover, it has been suggested that the extent to which individuals are motivated by social norms depends on the behaviour being studied; this may be different for health‐risk behaviours (e.g., smoking) versus health promoting behaviours (e.g., physical activity) [64, 81]. Another limitation was that only current physical activity behaviour was measured. As such, temporal relationships between the explanatory variables and PA behaviour could not be assessed and the associations should be interpreted as cross‐sectional rather than predictive.

We acknowledge that the lack of representation from non‐Western populations limits the study's contribution to addressing the geographic imbalance in health behaviour research. However, as this study was funded by the EU, participation was restricted to European partners within the research consortium. Inclusion rates differed across sites, resulting in lower sample sizes in some of the participating countries. This limits the interpretability and reliability of the country specific estimates, in the Swedish and Polish sample. Therefore, country specific findings should be interpreted with caution and should be considered strictly exploratory. Moreover, we acknowledge the importance of recognizing intracultural heterogeneity, also within cancer patient populations, which may not have been fully captured in the relatively homogenous pool from which we recruited across sites.

Amongst the strengths of this study are the use of a large and international sample, which supports the generalizability of the findings, as well as the rigorous statistical analysis (SEM), allowing for simultaneous estimation of multiple pathways. In addition, we evaluated the applicability of both the TPB with intention‐PBC interaction and the TPB, contributing to the evidence for‐ and understanding of the TPB in the context of cancer care.

### 4.3 Clinical Implications

The clinical implications of this study are broadly consistent with prior TPB‐based studies in cancer populations, which suggested that targeting attitudes, and perceived behavioural control could increase the success of interventions [13, 34]. However, it is worth noting that some constructs may be more susceptible to intervention than others. For example, informing patients of the health benefits of exercise (instrumental attitude) may be easier than persuading them they can overcome barriers (PBC) [82].

The current study highlights the TPB's relevance not only for theoretical understanding, but also as a foundation for developing effective intervention strategies to promote PA in patients with MBC. Specifically, our results emphasize the importance of addressing health‐related barriers to PA, either through reducing these barriers or overcoming them. Importantly, exercise in itself effectively reduces several of these health‐related barriers in this population, including pain, fatigue, or shortness of breath [10, 11, 12, 13, 14]. To optimize uptake and adherence, future PA interventions for this population should include strategies aimed at increasing self‐efficacy (PBC) through structured professional support.

## 5 Conclusion

In summary, our results indicate that the TPB is applicable for understanding and supporting physical activity behaviour in patients with MBC. We demonstrate and discuss how perceived behavioural control could be increased to overcome health‐related barriers in this population. Additionally, improving affective and instrumental attitude towards physical activity could enhance PA intervention outcomes.

## Author Contributions

**Esther de Jongh:** conceptualization, formal analysis, writing – original draft, writing – review and editing, visualisation. **Johanna Depenbusch:** conceptualization, resources, investigation, methodology, data curation. **Anne May:** funding acquisition, supervision, project administration, investigation, writing – review and editing. **Anouk Hiensch:** investigation, writing – review and editing. **Yvonne Wengström:** investigation, project administration, writing – review and editing. **Jon Belloso:** investigation, project administration, writing – review and editing. **Milena Lachowicz:** investigation, project administration, writing – review and editing. **Lonneke van de Poll‐Franse:** writing – review and editing. **Karen Steindorf:** conceptualization, project administration, writing – review and editing. **Martijn M. Stuiver:** conceptualization, project administration, formal analysis, software, methodology, validation, writing – review and editing.

## Funding

This study was part of the PREFERABLE project that has received funding from the European Union's Horizon 2020 research and innovation programme under grant agreement No 825677.

## Conflicts of Interest

The authors declare no conflicts of interest.

## Supporting information


Supporting Information S1

Supporting Information S2

## Data Availability

The data that support the findings of this study are available from the corresponding author upon reasonable request.
