# Supplementary material for: Understanding Physical Activity in Patients With Metastatic Breast Cancer: An Analysis Rooted in the Theory of Planned Behaviour
Source: Psychooncology. 2026 Apr 16;35(4):e70457. doi: 10.1002/pon.70457 (PMC13084298; doi:10.1002/pon.70457)

**Theory of Planned Behaviour: State of the Evidence**

The TPB posits that the closest determinant of a measured behaviour is *intention*, or an individual’s conscious objective to perform a behaviour (Ajzen, 1991). In the model, *intention* follows from *attitude*, *subjective norms*, and *perceived behavioural control* *(PBC).* These constructs may act together in their prediction of *intention*, and PBC can also contribute directly to the execution of the behaviour (Ajzen, 1991).

An individuals’ **attitude** towards the intended behaviour is composed of two subdomains. Instrumental attitude reflects if an individual considers the behaviour to be useful or serve the intended purpose, and affective attitude reflects an individuals’ affect towards the behaviour (e.g. feelings of enjoyment). This variable consistently correlates with health behaviour, yet it varies in strength among studies involving different cancer patient populations (Andrykowski et al., 2006; Min et al, 2022; Karvinen et al., 2008).

**Subjective norm** indicates to what extent the (dis)approval of others plays a role in mediating an individual’s behaviour, often measured through normative beliefs of others (injunctive and descriptive norms), and the motivation to comply with them (Ajzen, 1991; Cialdini et al., 1991). Although demonstrated in some studies, evidence for the role of subjective norms in predicting intention or behaviour is weaker than for PBC and attitude, and even absent in some (Jaganathan et al., 2023; Trinh et al., 2012; Li et al., 2025; Dumitrescu, 2011; Jones, 2007).

**Perceived behavioural control** **(PBC)** reflects an individual’s perceived ability to perform an intended behaviour and the perceived degree of control over this behaviour, and many studies have found it to be a strong predictor of intention and subsequent performance (Hagger et al., 2022; Prapavessis et al, 2005; Bandura, 1997; Hagger et al., 2022). PBC is often operationalized through **self-efficacy**, as the two constructs are widely accepted to be aligned (Ajzen, 1991; Armitage and Conner, 2001). Besides the indirect correlation between PBC and behaviour through intention, evidence suggests there may also be a direct correlation between PBC and several health-related behaviours in populations of people living with or beyond cancer (Jones et al., 2005; Buffart et al., 2018; Min et al., 2022).

Ajzen's original model (figure 1) proposes that PBC moderates the relationship between intention and behaviour, yet studies have moved away from assessing this since then. Barriers are often considered to be a component of PBC, as is the case in Ajzen’s model. However, specifically in the context of patients with MBC, it is of interest to study which barriers are important and how these mediate the TPB pathways.

*[Insert Figure 1 here]*

**Figure 1.**

The original model of the TPB as conceived by Ajzen (1991).


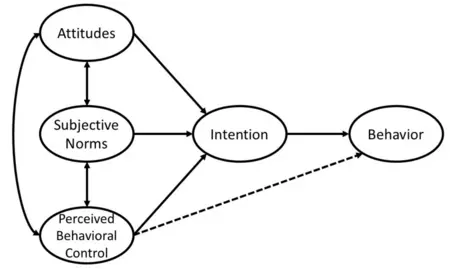

Supplement: Supplementary file 1 — Supporting Information S1 [file PON-35-e70457-s001.docx]
