# Supplementary material for: Understanding Physical Activity in Patients With Metastatic Breast Cancer: An Analysis Rooted in the Theory of Planned Behaviour
Source: Psychooncology. 2026 Apr 16;35(4):e70457. doi: 10.1002/pon.70457 (PMC13084298; doi:10.1002/pon.70457)
Supplement: Supplementary file 2 — Supporting Information S2 [file PON-35-e70457-s002.docx]

| **Construct** | **Measure/Instrument**  **(Source if applicable)** | **Items** | **Response scale** | **Reliability (α)** |
| --- | --- | --- | --- | --- |
| **Instrumental attitude** | Rating of six cognitive and affective aspects on a 7-point likert scale  (Speed-Andrews et al., 2014) | *Please choose the response category that best applies to you.*   1. *Useless - Useful* 2. *Harmful - Beneficial* 3. *Unwise - Sensible* | -3 – 3 | 0.83 |
| **Affective attitude** |  | 1. *Unpleasant - Enjoyable* 2. *Boring - Fun* 3. *Difficult - Easy* | -3 – 3 | 0.79 |
| **Perceived behavioural control (PBC)** | Exercise Self-Efficacy scale  (Resnick & Jenkins, 2000) | Imagine that you regularly try to exercise. How confident are you that you will exercise in the following situations?   1. *When I feel tired* 2. *When I am in a bad mood or feel depressed* 3. *When I feel stressed* 4. *When I am busy and pressed for time* 5. *When I have nobody to exercise with* 6. *When I don’t feel that exercise is helping to improve my physical fitness* 7. *When I feel stiff and sore* 8. *When the weather outside is bad* 9. *When I am not enjoying the exercises* | 0 – 4  Not at all confident – extremely confident | 0.89 |
| **Subjective norm** | Developed by authors | *I would exercise if my doctors and other health care professionals encourage me to do so.* | 0 - 4 | N/A (single item) |
| **Intention** | Developed by authors | 1. *Did you intend to start or continue exercising in the month prior to the coronavirus outbreak?* | 0 – 6  Definitely no – Definitely yes | N/A (single item) |
| **Barriers** | Possible barriers were rated on a five-point likert scale  (Cadmus-Bertram et al., 2020; Gustaw et al., 2017) | To what extent do the following reasons stand in your way for exercising on a regular basis?   1. *Feeling to weak to exercise* 2. *Health conditions other than cancer* 3. *Pain* 4. *Shortness of breath* 5. *Tiredness* 6. *Fear of falls or injury* 7. *Being unsure how to get started* 8. *Being unsure how much exercise I should do* | 0 – 4  Not at all – very much | 0.83 |
| **Behaviour** | Self-developed: 4 items based on the Godin Leisure Time Questionnaire  (Godin & Shephard, 1985) | Total minutes of at moderate and vigorous intensity PA during a week | Days per week and minutes per day  (for each exercise intensity category) |  |

Appendix A. Table of measures
